# What happens to the external carotid artery following carotid endarterectomy?

**DOI:** 10.1186/1471-2482-8-20

**Published:** 2008-11-25

**Authors:** Saleh M Abbas, David Adams, Peter Vanniasingham

**Affiliations:** 1 Middlemore hospital; Department of Surgery, Auckland, New Zealand

## Abstract

**Aims:**

The effect of carotid endarterectomy on the patency of the external carotid artery is unknown. We conducted a retrospective study to evaluate the long-term changes in the external carotid artery following carotid endarterectomy.

**Methods:**

Data was prospectively recorded for all patients who had carotid endarterectomy between 1997 and 2006 in our vascular surgical unit. These patients had follow-up with carotid duplex ultrasound to assess the patency of the internal and external carotid artery. The data were retrieved and evaluated for patency and flow characteristic in both arteries before and after surgery.

**Results:**

Carotid endarterectomy was performed on 255 occasions in 236 patients over the ten year study period. Immediate and long-term outcome of carotid endarterectomy is comparable to results at other major centers. Stenosis of the external carotid artery as detected by duplex scan occurred in 5.5% of patients and was totally asymptomatic.

**Conclusion:**

Our results of carotid endarterectomy are comparable to other centers. Long-term follow up of the external carotid artery with duplex scan showed asymptomatic stenosis in a small percentage of patients.

## Background

During carotid endarterectomy, surgeons often perform avulsion endarterectomy to the external carotid artery. There is limited data supporting the routine use of this technique. The aim of this study was to evaluate the long term anatomical changes that occur to the external carotid artery following carotid endarterectomy[1].

Trans-catheter embolization and ligation the external carotid artery is often undertaken to cut the blood supply of large hypervascular tumours, and for bleeding in severe epistaxis and trauma [2]. This procedure is safely performed with no ischaemic consequences to tissues in the head and neck region.

Previously some surgeons routinely performed endarterectomy of the external carotid artery during carotid surgery. However lack of data to support the necessity of that prompted vascular surgeons to do only limited external carotid endarterectomy regardless of its degree of stenosis[1].

The anastomosis between the middle meningeal artery (a branch from the maxillary artery) and the ophthalmic artery is a well described anatomic feature but the incidence is not known [3-5]. In rare cases, the main contribution to the ophthalmic circulation is from the middle meningeal artery. This variation in origin of the ophthalmic artery can result in catastrophic consequence following occlusion of the external carotid artery [4].

Pathologic changes in the external carotid artery following carotid endarterectomy are not well studied. Ascer et al [5] reported data on 114 carotid endarterectomies and concluded that avoiding external carotid endarterectomy during standard carotid endarterectomy does not result in significant progression of external carotid artery stenosis or occlusion.

## Methods

The Hospital electronic data system was searched for patients who had carotid endarterectomy between January 1997 and December 2006. Patients were included if they had carotid endarterectomy performed for symptomatic internal carotid artery stenosis that was more than 70%. Prospectively recorded data was reviewed retrospectively about these patients and included demographic details, presenting symptoms, duplex ultrasound scan, surgery outcome, complications, and the presence of contralateral carotid artery disease.

All patients were followed up with duplex ultrasound imaging at regular intervals to monitor the patency and flow characteristics of blood flow in the internal carotid artery, common carotid and external carotid arteries. The duplex ultrasound was usually performed at 6 weeks following surgery, and then at three monthly intervals for a year and six monthly intervals for up to five years. The occurrence of stroke or transient ischaemic attack during follow up was recorded from the hospital data system.

## Procedure

Internal Carotid endarterectomy was performed in symptomatic patients who had severe carotid artery stenosis (≥ 70% stenosis involving the carotid artery bifurcation). This was established by duplex ultrasound imaging. None of the external carotids had stenosis before surgery. Preoperative cardiac risk was assessed, and cardiac investigations undertaken as appropriate.

Operative procedures were performed under general anaesthesia. Symptomatic patients who had recurrent transient ischaemic attacks were treated with heparin preoperatively. Prior to carotid clamping, all patients were given a therapeutic dose of heparin (100 unit per Kg body weight). All carotid endarterectomies were performed by longitudinal arteriotomy through the common and internal carotid arteries. Selective carotid shunting was used in patients who had an internal carotid artery back-flow pressure of less than 60 mmHg. Temporary shunt was also used for patients who had recently had a stroke.

The arteriotomy was closed primarily or with synthetic patch angioplasty. Anticoagulation was generally not reversed with protamine. Patients were continued on aspirin in the immediate postoperative period.

## Results

There were 255 carotid endarterectomies on 236 patients (143 males). The median age was 72 years (range 52–92). Median hospital stay was 3 days. Mean follow up with duplex ultrasound scan 3 years (95% confidence interval 2.7–3.3).

One patient died in hospital due to postoperative intracerebral hemorrhage, which was preceded by hypertension. Hypertension that required intravenous vasodilator in the intensive care unit was seen in 30 patients (12%); one of these patients had severe confusion, and another had grand mal seizures. Acute myocardial infarction (defined as raised cardiac troponin with or without ECG changes) complicated 11 (4.3%) operations; two of these patients developed atrial fibrillation and one developed acute left ventricular failure.

Three Patients had to return to theatre after developing neurological symptoms; two were found to have occluded internal carotid arteries, whilst the third had patent endarterectomy site. All three had minimal residual disability.

Temporary dysfunction of the hypoglossal nerve (that usually manifested as dysarthria) occurred in seven (2.7%) patients. Three patient had injury of the recurrent laryngeal nerve, of which two patients made full recovery (Table 1).

**Table 1:** Complications of carotid endarterectomy (255 procedures)

Complication	Number	Percentage
Death	1	0.4%
Stroke	3	1.2%
TIA	7	2.7%
Myocardial infarction	11	4.3%
Hypertension	30	11.7%
Recurrence of stenosis	30	11.7%
Hypoglossal nerve damage	7	2.7%
Recurrent laryngeal nerve damage	1	0.4%

Duplex ultrasound imaging of the external carotid artery showed new stenosis (>50%) in 14 patients (5.5%). Stenosis was considered moderate if there was 50–69% reduction of the cross sectional area and severe when there was 70% or more reduction of the cross sectional area.

**Figure 1:**
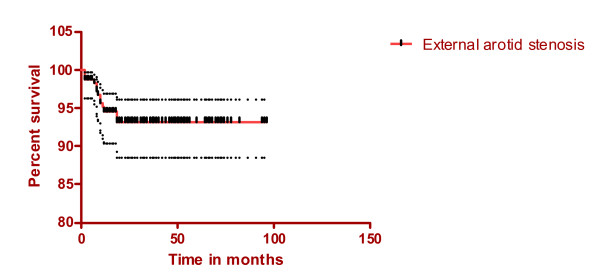
(Stenosis of the external carotid artery, mean and 95% confidence intervals).

The stenosis was 50–69% in five patients and more than 70% in nine patients; five of these had total occlusion of the external carotid artery. The mean time for the progression to stenosis of 50% or more was 9 months (median time was 9 months; quartile range 6 and 15 months). None of these patients reported any symptoms of ischaemia or ophthalmic symptoms (Figure 1).

**Figure 2:**
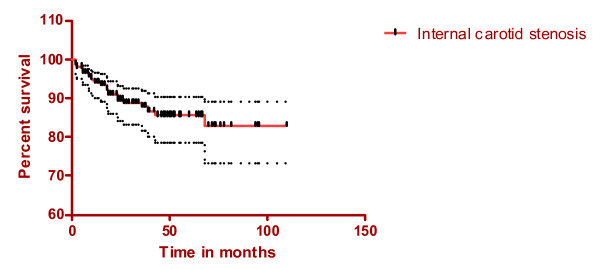
(Stenosis of the internal carotid artery, mean and 95% confidence intervals).

Restenosis (> 50% narrowing of the lumen) of the internal carotid artery occurred in 30 patients (11.7%), of which five were completely occluded. Median time for restenosis in the internal carotid artery was 15 months (quartile range 6 and 18 months). Seven of those recurrences were symptomatic (five had transient ischaemic attacks and 2 had strokes) and three patients required carotid stenting. Dilatation of the internal carotid artery was seen in two patients and was asymptomatic (Figure 2).

## Discussion

The results of carotid endarterectomy at our urban community hospital are comparable with reports from other western countries specialized vascular units [7,8]. The mortality and stroke rates is low at 0.4% 1.2% respectively. External carotid artery stenosis was asymptomatic; the frequency of this is comparable to the few other published studies [1,6,7]. Carotid endarterectomy remains a low-risk operation for the treatment of severe symptomatic carotid disease, with excellent long-term results and durability [9-11].

The rate of cranial nerve injury remains low and is comparable to other studies [10]. We had 11.7% recurrence of stenosis, which is comparable to other studies; however, those studies had shorter follow-up of 1–2 years and demonstrated rates of re-stenosis of 4% – 14% [12-14].

Archie et al [14] reported 9% of patients had severe (>70%) stenosis of the external carotid artery following carotid endarterectomy; and found that the repair of severely stenosed or occluded external carotid artery identified during carotid endarterectomy had a poor outcome, and suggested that technique and management of the external carotid artery during routine carotid endarterectomy needs further investigation and modification.

Occlusion of the external carotid artery is known to occur after carotid endarterectomy and is usually without consequences. In some individuals, however, the external carotid artery is the primary source of arterial blood flow to the eye via the middle meningeal artery [15,16]. Authors have therefore suggested that surgeons should avoid causing external carotid artery occlusion, by meticulously removing not only the ICA plaque, but also the entire external carotid artery plaque [17].

Ocular ischaemic syndrome is also known to occur in patients with bilateral occlusion of the external carotid arteries even in the presence of a patent common and internal carotid arteries. Unilateral ocular ischaemic symptoms can follow consecutive bilateral carotid endarterectomies. It can also be exacerbated after ipsilateral carotid endarterectomy in the setting of pre-existing contralateral external carotid artery occlusion.

Ricco et al described performing intraoperative completion arteriography following 605 carotid endarterectomies. This study demonstratedn stenosis in 114 cases; including 17 involved the internal carotid artery and 73 involving the external carotid artery [18]. Ascher et al described major technical defects using intraoperative duplex scanning (>30% luminal internal carotid artery stenosis, free-floating clot, dissection, arterial disruption with pseudo-aneurysm) were repaired. No clinically detectable postoperative thromboembolic events occurred in this series of 605 endarterectomies [8]. No similar data were available on the external carotid artery.

In conclusion this retrospective report shows carotid endarterectomy results in stenosis of the external carotid artery in a small proportion of patients, but this stenosis was totally asymptomatic.

## Competing interests

The authors declare that they have no competing interests.

## Authors' contributions

SA: Study design, gathering and analysis of data, writing of the manuscript

DA: Study design, review of the manuscript

PV: Review of the manuscript

## Pre-publication history

The pre-publication history for this paper can be accessed here:


